# Atomic Force Microscopy for Protein Detection and Their Physicoсhemical Characterization

**DOI:** 10.3390/ijms19041142

**Published:** 2018-04-10

**Authors:** Tatyana O. Pleshakova, Natalia S. Bukharina, Alexander I. Archakov, Yuri D. Ivanov

**Affiliations:** Institute of Biomedical Chemistry, 10, Pogodinskaya St., 119121 Moscow, Russia; t.pleshakova@gmail.com (T.O.P.); natalie_buharina@list.ru (N.S.B.); alexander.archakov@ibmc.msk.ru (A.I.A.)

**Keywords:** atomic force microscopy, protein fishing, protein characterization

## Abstract

This review is focused on the atomic force microscopy (AFM) capabilities to study the properties of protein biomolecules and to detect the proteins in solution. The possibilities of application of a wide range of measuring techniques and modes for visualization of proteins, determination of their stoichiometric characteristics and physicochemical properties, are analyzed. Particular attention is paid to the use of AFM as a molecular detector for detection of proteins in solutions at low concentrations, and also for determination of functional properties of single biomolecules, including the activity of individual molecules of enzymes. Prospects for the development of AFM in combination with other methods for studying biomacromolecules are discussed.

## 1. Introduction

Study of intermolecular interactions and a quantitative characterization of the mechanisms of those interactions is of importance for profound understanding of different biological processes. In the past decades, novel techniques have been developed to manipulate and study single biomolecules [1,2]. The ability to monitor biological processes at this fundamental sensitivity level provides a better understanding of the molecular mechanisms. Averaging over an ensemble, distribution and fluctuation of molecular properties may be excluded from consideration at the single-molecule level. Thus, single-molecule experiments allow to study properties of heterogeneous systems compared to the standard ensemble methods [2]. Atomic force microscopy (AFM) occupies a prominent place in the range of techniques operating at the single-molecule level.

AFM was developed as a method for visualizing objects in 1986. In this method, three-dimensional (3D) topography images and structural details of the samples are obtained using cantilever which scans a surface [3]. It has become a powerful tool in the field of biomolecules and cells study due to its ability to scan a surface with (sub-) nanometer resolution in liquid [4]. Moreover, AFM does not require dye or fluorescent labels as optical microscopy. It allows for the imaging of biological objects from single molecules to the living cells as well as their manipulation. A lot of AFM modes have been developed to study biomolecules, and those modes are widely described in literature, but there is still a lot of research focused on improvements in AFM electronics, experiments with cantilever size [5], data interpretation and so on. This review is focused on the AFM capabilities to study protein biomolecules, their physicochemical properties, and perspectives to adapt AFM for biomedical detection of the low-abundance target proteins in solution.

## 2. Atomic Force Microscopy (AFM) Visualization of Proteins

**Figure 1:**
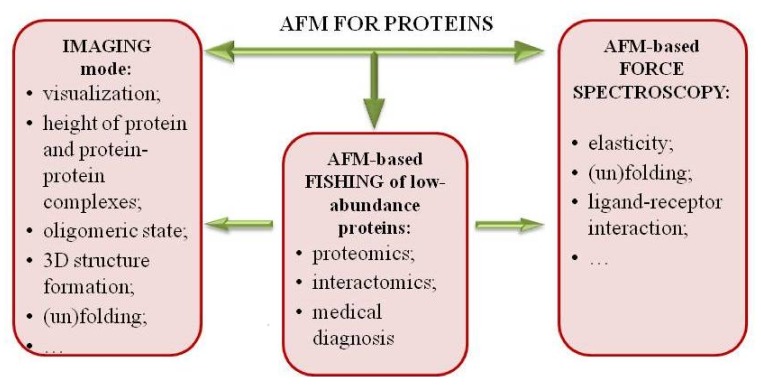
Application of atomic force microscopy (AFM) to study proteins. It includes three major aspects such as imaging mode, AFM-based force spectroscopy and AFM-based fishing of low-abundance proteins. Each of them has a variety of possibilities to investigate physicochemical properties of proteins.

AFM has a great potential for biology applications with the broad range of visualization techniques (Figure 1, left column). Contact and dynamic modes have been the most popular ones over the years, and such modes as multiparametric and multi-frequency imaging, molecular recognition and high-speed AFM have been developed recently.

In a contact mode, AFM probe raster scans a sample surface at a constant setpoint. Constant force between probe and surface or constant height may be chosen as a setpoint. Resolution of the obtained topography image depends on a probe radius, sample roughness, its physical properties, and tracking accuracy of the feedback loop (performance).

A lot of biological objects such as animal cells [6,7], membrane surface of cell and membrane proteins [8,9], DNA and RNA [10,11,12], lipid films [13,14,15] were visualized by commercial AFMs. If objects have small height difference, for instance, proteins with height of 1 nm over the membrane surface, lateral and vertical resolutions of topography image may be less than 1 nm and 0.1 nm, respectively [16,17,18,19]. Vertical resolution of AFM is about 0.1 nm, which is close to the resolution of X-ray crystallography [1,20]. Thanks to high values of resolution and signal-to-noise ratio, it was possible to study the functionally dependent oligomeric state of water-soluble and membrane proteins [21].

The main disadvantage of contact mode is direct mechanical interaction between the probe and the surface. This could often lead to destruction of the sample surface during imaging [4,22]. Contact mode is widely used to specify properties of the solid substrates while to image the soft biological systems there should be an accurate adjustment of the force applied by cantilever. The force should be less than 100 pN to avoid a nonreversible deformation of the sample [23].

Dynamic mode of AFM imaging (tapping or oscillating mode) was developed to reduce friction and force between the tip and the sample. In this mode, cantilever is forced to oscillate close to its resonant frequency while contouring the sample [1,22]. The most popular dynamic modes use the oscillation amplitude of cantilever and its resonance frequency as feedback parameters because they change when the tip is near the sample surface [1,4,24,25]. There are also other variants of dynamic mode which use different signals as feedback or even induce cantilever oscillation at different frequencies [26]. In reality, interaction between the tip and the sample has quite a complex mechanism taking into account stiffness, roughness and surface charge of the sample. All those parameters could affect cantilever oscillation and change or invert topography contrast [26].

In an ideal visualization of soft biological objects, the AFM tip touches the sample surface at the very bottom of its downward movement. It helps to image objects which have a low adsorption on the support surface such as DNA, filaments and separate macromolecules of proteins [27,28,29]. Using AFM to study protein systems provided new insights into analysis of proteins [30,31], for example, measurements of structural and mechanical properties of single protein molecules in physiological buffers [32]; revealing the dynamic processes of single molecules including intermolecular [33,34,35,36,37,38] and intramolecular forces [39,40]; measurements of enzyme activity by detecting the motion of protein molecules [41]. AFM images of inorganic crystals have atomic resolution while resolution for biological objects is limited by submolecular level. The main areas of morphological AFM research of proteins have been formation of protein-nucleic acid or protein-protein complexes, oligomerization, and organization of protein molecules in biological membranes and Langmuir films [42,43].

Recent studies of water-soluble and membrane proteins revealed their submolecular details. Improvements in sample preparation [44,45] and visualization techniques [17,46] provided possibilities of imaging at subnanometer resolution. In the case of water-soluble proteins, image resolution is lower than one for membrane proteins which can form two-dimensional (2D) crystal layers [47].

AFM is widely used to measure heights and molecular volumes of protein molecules immobilized onto a support surface revealing their linear correlation with molecular weight [29,48,49,50,51].

**Figure 2:**
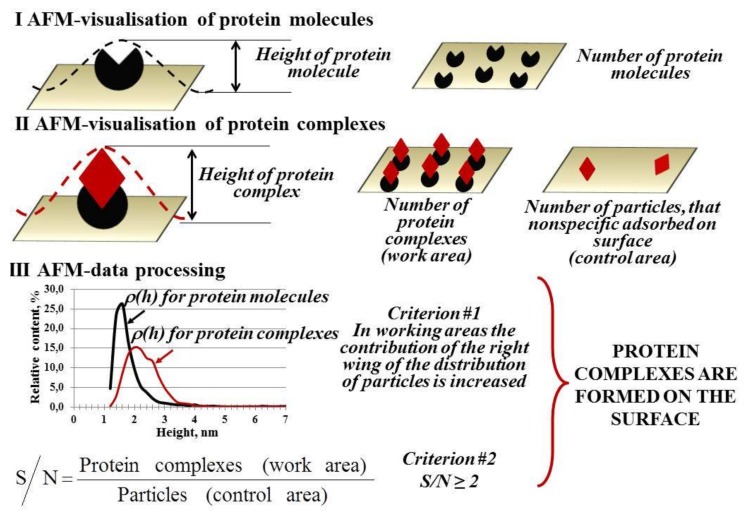
Sequence of stages of AFM measurements and data processing for obtaining information on stoichiometric parameters of protein macromolecules and protein complexes. At step (**I**), during the visualization by AFM, the height of biomolecules—components of protein complexes adsorbed (immobilized) on the support surface—is determined. At step (**II**), visualization of the surface after the incubation of the support in a solution of partner protein is carried out, and the height of the complexes is determined. At step (**III**) of processing of AFM data, relative distributions of the visualized objects with height *ρ*(*h*) are plotted, and the number of these objects per unit area is calculated. If the data obtained meet the criteria #1 and #2, the formation of protein complexes on the support surface is confirmed.

Measuring the heights and molecular volumes of protein molecules allows to monitor a formation of protein complexes. Figure 2 presents a scheme of this monitoring process.

For instance, individual hepatitis C virus core-antigen (HCVcoreAg), antibodies against the hepatitis C virus core antigen (anti-HCVcoreAg) and their complex had significantly different heights of (1.5–2.0) nm, (1.5–2.0) nm and (5.0–7.0) nm, respectively [52].

An important application of AFM is studying the oligomeric state of proteins which may be useful for understanding the mechanisms of their functions. For example, cytochrome P450-containig monooxygenase systems oxidize a broad variety of endogenous and exogenous substrates thus they remain a high research interest [53]. The enzymatic P450 systems may be conventionally divided into two major groups: (I) single-protein self-sufficient systems (P450 102A1) [54], which do not require protein partners for the catalytic reaction to occur [55] and (II) more complicated, multiprotein systems (P450 101 [P450cam] responsible for camphor degradation, P450 11A1 [P450scc] responsible for cholesterol conversion to pregnenolone and P450 2B4 [P450LM2]), when catalytic reactions involve interaction with a protein partner [53,56,57,58]. The information on the enzyme’s structure is very important for elucidation of the reaction mechanisms of (I) and (II) groups. AFM was capable of visualizing and obtaining the height of protein molecules and protein-protein complexes forming in those systems [59,60,61,62,63,64,65,66,67,68] in a tapping mode. Analysis of the density distribution with height *ρ*(*h*) and its approximation allowed for the determination of parameters of single proteins as well as protein complexes.

AFM allowed to register formation of binary and ternary protein complexes in all the three P450101, P450 11A1 and P4502B4-containing monooxygenase systems [59]. For P450 101-containing system, AFM allowed to visualize the individual proteins PdR, Pd and P450 101 on mica and measure their heights [67,69]. The heights of proteins were estimated as (2.6 ± 0.3) nm for (P450 101), (2.0 ± 0.3) nm (Pd) and (2.8 ± 0.3) nm (PdR) [67]. The fact that the actual sizes of proteins are somewhat less than those obtained by X-ray crystallography or nuclear magnetic resonance spectroscopy was explained by the molecule contraction under the force applied by AFM tip. The binary PdR/Pd, Pd/P450 101 and PdR/P450 101 complexes were identified having their characteristic heights of (4.9 ± 0.3) nm, (4.3 ± 0.3) nm and (5.1 ± 0.3) nm which were greater than those of single proteins. Along with the binary complexes, the ternary PdR/Pd/P450 101 complexes were also registered. Accordingly, the characteristic heights of ternary PdR/Pd/P450 101 complexes were in the range of (6.5–9.5) nm that was greater than those of binary complexes. The approach for obtaining the AFM images of isolated membrane proteins Fp, 2B4 and b5 and their complexes involved monomerization procedure in solution with the use of Emulgen 913 at the first step followed by AFM registration and counting of proteins at the second step [65]. As was demonstrated during the course of this procedure, the membrane proteins became monomers while enzyme molecules retained their activity. Thus, the separately located Fp, 2B4 and b5 were visualized with the heights of (2.2 ± 0.2) nm, (2.3 ± 0.2) nm and (1.8 ± 0.1) nm, respectively. The binary Fp/2B4 and 2B4/b5 complexes were higher than isolated proteins: their characteristic heights were in the range over 2.7 nm, with respective local maximums of (3.4 ± 0.2) nm and (2.8 ± 0.2) nm [65]. No formation of Fp/b5 complexes was registered. The ternary Fp/2B4/b5 complex heights were found to be in the range of (4.3–6.2) nm.

AFM were used to monitor cytochrome CYP11A1 monomerization in solution without phospholipids [69]. It was shown that the incubation of CYP11A1 with 12% Emulgen 913 led to dissociation of hemoprotein aggregates to monomers with the monomerization degree of (82 ± 4)%. Despite the monomerization procedure, CYP11A1 remained its functional activity. AFM was employed to detect and visualize the isolated proteins as well as complexes formed between the components of the cytochrome CYP11A1-dependent steroid hydroxylase system. Both Ad and AdR were present in solution as monomers. The typical heights of the monomeric AdR, Ad and CYP11A1 were (1.6 ± 0.2) nm, (1.0 ± 0.2) nm and (1.8 ± 0.2) nm, respectively. The binary Ad/AdR and AdR/CYP11A1mon complexes with the heights of (2.2 ± 0.2) nm and (2.8 ± 0.2) nm, respectively, were also registered by AFM. In addition, the ternary AdR/Ad/CYP11A1 complexes with a typical height of (4.0 ± 1.0) nm were imaged.

Oligomerization degree was estimated for CYP102A1 protein using AFM visualization [60]. Immobilized protein molecules were visualized as monomers and oligomers. Objects with the height of (2.2 ± 0.1) nm were referred to as monomers, objects with the height of (3.4 ± 0.7) nm were referred to as oligomers. Share of objects was (48 ± 10)% and (52 ± 10)%, respectively. It is known that the oligomeric form of CYP102A1 has a higher catalytic activity than its monomeric form [54], and AFM allowed to estimate directly the ratio between those forms.

In neurodegenerative disease studies a novel, on-surface aggregation pathway of amyloidogenic polypeptide was discovered. AFM was used to demonstrate directly that on-surface aggregation took place at a concentration at which aggregation in solution was not observed. The experiments were performed with the full-size β-amyloid peptide (Aβ42), a decapeptide Aβ (14–23) and α-synuclein; all three systems demonstrated a dramatic preference for the on-surface aggregation pathway compared to the aggregation in the bulk solution. Time-lapse AFM imaging in solution have shown that over time, oligomers grew in size and number and released in solution, suggesting that assembled aggregates can serve as nuclei for aggregation in bulk solution [70].

AFM ability to determine protein oligomeric state may be extended to test its activity. For instance, it was demonstrated that monoclonal antibodies were self-assembled into hexamers, which formed 2D crystals in aqueous solution. Direct observation of antibody–antigen interactions using frequency modulation atomic force microscopy (FM-AFM) revealed that immunoglobulin G (IgG) molecules in the crystal retained their immunoactivity [29].

An approach to measure the activity of single oligomers of the hemecontaining enzyme cytochrome P450 CYP102A1 (CYP102A1) by atomic force microscopy (AFM) has been demonstrated [63]. It was found that the amplitude of fluctuations of the height of single CYP102A1 molecules performing the catalytic cycle is twice as great as the amplitude of fluctuations of the height of the same enzymes in the inactive state. It was shown that the amplitude of height fluctuations of a CYP102A1 protein globule depends on temperature, the maximum of this dependence being observed at 22 °C. The activity of a single CYP102A1 molecule in the unit amplitude of height fluctuations of a protein globule per unit time was 5 ± 2 Å/s.

Limitation of standard AFM modes to study biomolecular processes is scanning speed. So, a high-speed AFM was developed to gain insights into mechanisms of molecular actions [71,72]. High-speed AFM was used to investigate the aggregation of α-synuclein protein. α-Synuclein is the major component of the intraneuronal inclusions called Lewy bodies which are the pathological hallmark of Parkinson’s disease. This protein may self-assemble into many different species such as soluble oligomers and fibrils. A detailed understanding of these structures and their relationship with the different aggregation steps is lacking, while it can provide insight into the pathogenic mechanism of Parkinson’s disease [73]. So, high-speed AFM has great potential but it is worth noting that experimental settings should be chosen carefully to not affect the results. It was shown that CYP102A1 deformation in a tapping mode with high-speed probes was greater compared to that in a tapping mode with standard probes. Increasing deformation led to the disappearance of CYP102A1 fluctuations during AFM monitoring of enzyme catalytic cycle [61].

Protein adsorption at solid-liquid interface has been a key topic in biomedical studies of biosensors and biochips [74], biomaterials [75,76], etc. Interaction force between protein and impervious surface depends on 3D protein structure and surface chemistry. Besides multiple studies on protein adsorption, it is still underexplored. A lot of methods based on different principles including infrared spectroscopy, Raman and circular dichroism spectroscopy, surface plasmon resonance spectroscopy, scanning electron microscopy and others has been engaged to study the interfacial properties of adsorbed proteins. However, all those methods provide averaged characteristics of biomolecules, while AFM allows to image adsorbed individual protein molecules under close to physiological conditions.

There is a high demand to overcome challenges of molecular and cell biology especially of drug design, which requires to study oligomerization, supramolecular structure and structural functions of membrane proteins in their native environment. Although only 30% of proteins encoded by the human genome are membrane proteins, they make 50% of all drug targets because of their stimulating and controlling functional activities of every life process [77]. Well-characterized membrane proteins have become convenient objects for AFM imaging at the beginning. One of the first studies was AFM visualization of native purple membranes of *Halobacterium halobium* [78] and visualization of 2D lattice of bacteriorhodopsin in them [79]. AFM allowed the identification of individual bacteriorhodopsin molecules on the purple membrane surface and revealed subnanometer features such as contribution of polypeptide chains to structural stability as barriers of protein unfolding [80,81]. Purple membranes adsorbed on mica were visualized in buffer solution by AFM, and it was found that its thickness depends on pH value of solution. Topography images of the cytoplasmic and extracellular surface also showed the same hexagonal lattice of bacteriorhodopsin [81]. Besides, a careful choice of experimental conditions increased a resolution of diffraction patterns up to 0.7 nm which gave an opportunity to resolve the structure of photoreactive unit with distance between its domains of 1.45 nm [42]. AFM observation of working ATP synthase allowed to image its 14 subunits rather than 10–12 ones predicted before [82]; that was the evidence of a submolecular level of AFM protein study.

## 3. Force Spectroscopy Mode

AFM is used not only for imaging. Force spectroscopy (FS) mode for investigation of elastic properties of protein molecules, which can be estimated from force distance (FD) curves obtained during the compressing stretching protein molecules under the probe force with the common setpoint limit of ~1 nN [1,83]. AFM-based force spectroscopy (AFM-based FS) became one of the fundamental methods in the surface chemistry, biochemistry and materials science (Figure 1, right column).

AFM-based FS allows to determine and quantify the biophysical properties of proteins. Such properties can be obtained from the single FD curves recorded in the approach-retraction cycle of the probe movement towards and away from the sample, respectively [1,84]. The approach part of the curve is used to measure height, surface forces and mechanical deformation of the sample, and to estimate its elastic modulus and energy dissipation. The retraction part of the curve is used to estimate adhesion forces. To measure all these sample properties, it is required to have high-accuracy control of tip-sample interaction, and tip geometry and its surface properties should be known.

Force volume (FV) imaging combines topographic imaging and FD curves recording at any point for further processing [1,84,85]. FV mode allows to quantify elastic modulus along to other parameters, but it has insufficient resolution and low rate. Besides, results can be interpreted only after scan data processing and calculation of Young’s modulus of the sample.

Among various models, Herz model has been widely applied to describe tip-surface contact omitting adhesion forces and to extract sample Young’s modulus but it has lots of limitations for proteins [86]. Herz model was developed for a semi-infinite elastic sphere of homogenous and isotropic materials, and proteins are nonhomogeneous and nonisotropic. Despite that it has been used to obtain numerical measure of protein elasticity [61,87].

One of the AFM-based FS modifications is a pulsed force mode when only the part of FD curve of tip pulling off the surface is recorded [88]. Lift mode is a two-passed method to measure the interaction forces while keeping constant the distance above the surface [85]. It is carried out after obtaining a standard topography image. It is also worth noting such abilities of various AFM-based FS modes as mapping the distribution of interaction forces automatically [22] and determining the local stiffness of materials (force modulation mode) [89].

In addition to real-time visualization of biological structures, AFM is used for direct measurement of adhesive forces between ligand and receptor. AFM-based FS allow to characterize individual interactions and complex formation instead of conventional studies of molecule population. AFM study of processes combining several different reactions (heterogeneity of interacting molecules, polyvalent binding or different combinations of complexes) may be conducted. The possibility of controlling the pull-off rate of cantilever, and thus the rate of breaking the complex of interacting partners, is an essential feature of AFM that allows for the estimation of the rupture forces along to kinetic and thermodynamic parameters of breaking the complex [2]. Different theoretical models are used to interpret AFM-based FS data and to convert values of rupture force and rate to thermodynamic and kinetic parameters of the reaction such as kinetic dissociation constant, Gibbs free energy, enthalpy, and entropy. The advantage of AFM is to measure the kinetic dissociation constant, which is not affected by reassociation events of ligand-receptor complexes occurring at the same time. Using AFM to study polyvalent interactions seems quite promising although it is a challenging task due to the complex stoichiometry of reaction. It is noteworthy that sample immobilization is necessary for AFM imaging, including covalent bonding, which is obviously one of the limitations of AFM.

The ability of AFM-based FS to measure interaction forces between ligand and receptor was demonstrated in the study of streptavidin-biotin pair under physiological conditions [90,91]. For the first time, interaction forces were measured between two surfaces functionalized with biotin, surfaces functionalized with streptavidin and biotin, and surfaces functionalized with biotin and biotin-streptavidin complex. In order to do that, attached to the AFM tip glass microsphere and mica surface were functionalized by biotin and streptavidin with covalent binding to bovine serum albumin (BSA) as a linkage. BSA have been non-specifically and irreversibly adsorbed onto glass and mica surfaces beforehand. There were not any residual forces between two biotinylated surfaces which made them suitable for measurement of biotin-streptavidin interaction forces in the piconewton range [42]. So far, adhesion forces between variety of ligand-receptor pairs such as biotin-avidin pair [92], antibody-antigen pair [93,94], between cell recognition proteins [95,96] and others were measured by AFM. Moreover, it was shown that the value of adhesion force was not constant but it was dependent on experiment dynamics [92,97,98,99].

Almost all protein chains fold into a unique 3D structure which is well-organized and biologically active despite having a linear sequence of amino acids in a protein synthesis. Simple calculations provide the evidence that searching for unique native conformation, even for a small protein containing 100 amino acid residues, will occupy a million years using random conformational search. Therefore, mechanisms of folding and unfolding of protein are important topics of structural biology and physics. Stretching force curves can be obtained with the following procedure: AFM tip approaches a protein on the surface, touches it and retracts back stretching a protein [1,19]. This kind of AFM-based FS demonstrated that different proteins can be unfolded reversibly to reveal the essential properties of protein folding. A stretching technique was also implemented for unfolding of the single protein domains [100,101]. In this case differences in force values, which are required to unfold the molecule domains, can be quantified as well as compared to the folding topology; stability of secondary structural features can be analyzed; the rate constant of unfolding the single molecule can be estimated [19].

The obvious advantage of AFM to study protein (un)folding is that transitional steps of the process become visible. Combination of high-resolution AFM and single-molecule FS was applied to study the unfolding of single bacteriorhodopsin molecules from native purple membrane at various physiological temperatures and to obtain detailed understanding about stability of individual structural elements [102].

It was demonstrated that data obtained by FS experiments of protein (un)folding are comparable in quality and quantity to data obtained by standard techniques. Mechanical unfolding of single protein molecule by AFM is considered to have the same steps as the unfolding process observed by traditional folding techniques but the significant advantage of using AFM is the capability to determine the reaction coordinate and to reveal unique unfolding events which could not be found without chemical denaturants [103]. However, there is also a contrary opinion that mechanical unfolding of protein by AFM exposes an autonomous mechanism which depends on the direction of applied extension [104].

Modern FS-based AFMs can record over 100,000 FD curves, each of them describes local physical properties and interaction; such a map may be compared to topography image of a sample. A variety of FS-based modes have been developed to characterize sample properties during imaging [105]. Implemented to AFM device from NT-MDT (Zelenograd, Russia), point-to-point measurement tool allows simultaneous registration of sample topography image and its physical properties such as elastic, electrostatic, magnetic and other properties [85]. The main feature of the method is the absence of lateral forces because AFM probe moves off contact with the sample surface in lateral direction; this is crucial factor for the soft and weakly adsorbed objects. Registration of the force signal is conducted the same way as in contact mode when cantilever deflection is a feedback signal which is proportional to a normal force. Another tool implemented to AFM from Bruker (Santa Barbara, CA, USA) is Peak Force Tapping (PFT) mode [106]. This mode operates as tapping mode, but cantilever oscillates at low frequency of about 1 kHz in a non-resonant mode with recording FD curve at every contact point with the sample surface. In PFT mode, topography image is obtained along with maps of adhesion force, energy dissipation, deformation and Young’s modulus of sample. If cantilever was calibrated beforehand then all parameters would be mapped and quantified during imaging (Quantitative Nano-Mechanical PFT). PFT mode has an essential benefit of direct control of tip-sample interaction allowing to apply really low visualization forces to study biological objects.

There are two parameters of FS-based AFM which have a great influence on design and results of AFM experiments [105]. One of them is lateral resolution, which depends on the tip radius, the sample drift, distance dependence of the tip-sample interaction, imaging force and the properties of the biological sample. Another is the temporal resolution which depends on number of pixels recorded on the AFM image and the rate of obtaining FD curve. Development of new oscillation modes [107], improvement of piezo elements and feedback loops are used to increase AFM resolution. So modern FS-based AFM is able to obtain maps of 512 × 512 pixels (pxls) of different characteristics of biological objects under native conditions at resolution close to 1 nm in the range of time intervals of 15–30 min [105].

Besides FS and FV imaging, a technique called Molecular recognition was developed for mapping specific interaction between two molecules simultaneously with topography imaging [108,109,110]. Interaction occurs between a molecule attached to the AFM tip (chemical modification or immobilized ligand) and a molecule attached to the substrate surface (immobilized on the surface or localized in biological object, i.e., cell membrane). Topography and molecular recognition images (AFM-based TREC) can be compared to determine correlation of binding events and topographic profiles [111,112,113]. A variety of biomolecular systems have been studied using TREC imaging such as antigen-antibody [114], antibody-protein [115], cells [116,117], and other ligand-receptor pairs. For example, in [118], recognition of anti-gp120 aptamer was done using envelope glycoprotein gp120 of human immunodeficiency virus (HIV-1) as the probing molecule attached to the tip [110].

Recognition imaging has a lot of biomedical potential to study specific receptor binding sites on the cells [116] or even explore tissue samples [119].

In TREC mode, tip is functionalized by the probing molecule using flexible linkers [120] such as polyethylene glycol (PEG), DSP and so forth. Functionalized probe is oscillated with amplitude in the range of 5–10 nm during imaging and every recognition event causes reduction of the amplitude value. Imaging time in this case is comparable with standard imaging in a tapping mode. However, because FD curves are not registered, there is no quantitative characteristics of binding events, but only their localization can be obtained [110].

Binding specificity of functionalized tip with a sample is another problem that occurred during TREC imaging. Experimental design should be developed to identify specific interaction between ligand immobilized onto the tip and target receptor on the surface. Thus, control experiments should be conducted when undesired ligand-receptor interactions are blocked with chemicals or mutant cells without any specific recognition sites. Recognition imaging of living cells can be challenging because AFM probe can be easily contaminated during study. Weakly adsorbed objects may change the geometry of functionalized tip that would lead to inaccurate imaging. In this case design of experiment should include initial imaging and analyzing of sample by non-functionalized tip.

## 4. AFM-Based Molecular Detector of Low-Abundance Proteins

AFM provides the opportunity of protein visualization in a counting mode, which promises to use it as a molecular detector. Molecular detectors are a new generation of devices based on technologies and instruments which are used to detect single biomolecules in biological fluid. Proteomics and biomedical studies have a high demand for high-sensitivity analysis systems based on molecular detectors, and there are several reasons for that.

For diagnosis of cancer and viral diseases it was shown that a concentration detection limit (CDL) had to be lower than 10^−15^ M [121]. However, most modern high-performance methods of proteomics such as mass-spectrometry with one- and two-dimensional electrophoresis or liquid chromatography (1- and 2-DE/LC MS) has CDL in the range from 10^−12^ to 10^−14^ M [122,123]. Therefore, it is necessary to increase a concentration sensitivity of analytical systems by several orders of magnitude to detect a high variety of low-abundance functional proteins having potential to be disease markers [123,124,125]. In the range of protein concentration from 10^−12^ to 10^−14^ M, only one thousand protein species can be detected in plasma which is a small part of total presumable amount of several million proteins [122,125,126].

The second reason for increasing the concentration sensitivity is a limited volume of sampling material (serum or plasma). In proteomics studies, serum is appealed to be of significant interest to search for disease biomarkers. It becomes an ideal biological sample since it is an information medium of the proteins released by diseased tissue. Variation in protein amount in response to pathogeneses makes serum an attractive clinical trial material. Calculations showed that the sample volume has to be of order 1 μL for protein identification at concentration of 10^−8^ M in serum by mass-spectrometer (MS). It has to be of order 10 L for protein identification at concentration of 10^−15^ M that is obviously impossible in reality [123].

Analysis of serum proteome is also quite a challenging task because of the wide dynamic range of proteins. Detection of target analytes at low concentration involves some difficulties due to the presence of high-abundance proteins, high level of salt and other interfered compounds in serum [122,123].

AFM-based molecular detector combines a method itself and fishing technique (AFM-fishing) [127]. Fishing is a process of catching target proteins from solution using “bait” [128]. Molecular fishing is an adaptation of affinity enrichment of target molecules based on the specific interaction between immobilized ligand (bait molecule) and one or several assumed functional partners (captured molecule). Different molecules from small organic molecules to proteins and nucleic acids are able to be the bait molecules. Fishing technique have been implemented to a lot of biochemical methods.

AFM fishing (Figure 1, middle column) for detection of low-abundance proteins has two steps:

(1) fishing of biomolecules from a big volume of biological fluid onto a small surface (concentrating instead of conventional removal of high-abundance proteins by chromatography and electrophoresis used for protein matrix separation);
(2) high-sensitivity detection of caught molecules using AFM-based molecular detector (registering and counting the single molecules and molecular complexes).

**Figure 3:**
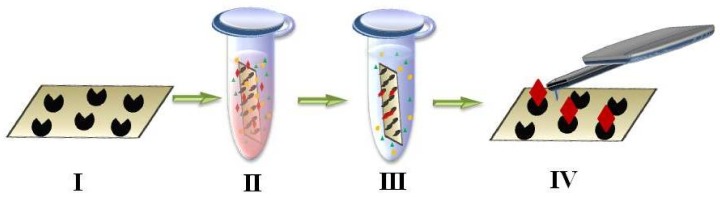
Experimental design of AFM-based fishing includes the following steps: (**I**) functionalization of the chip by immobilizing the bait molecules for further biospecific registration of protein complexes; (**II**) biospecific fishing of protein from solution during chip incubation in analyzed fluid; (**III**) chip rinsing to remove the components adsorbed nonspecifically onto the surface; (**IV**) AFM imaging in a counting mode to detect protein complexes.

During AFM-based fishing chip surface becomes bait area for concentrating the target proteins. Figure 3 presents the scheme of AFM-based fishing technique. Chip surface is functionalized by antibodies or aptamers in the case of biospecific analysis [122,123,127,129] (Figure 3, first step).

During chip incubation in analyzed fluid (Figure 3, second step) ligand-target complexes are formed on the chip surface functionalized by ligands. Chip surface is scanned by AFM to visualize (Figure 3, fourth step) and count formed complexes using the fact that the heights of complex components are smaller than the height of the whole complex. AFM-based fishing was applied to detect such serological marker as hepatitis C virus core-antigen (HCVcoreAg) in human serum using antibodies against the hepatitis C virus core antigen (anti-HCVcoreAg) as bait [52,130]. After incubation of AFM chip functionalized with anti-HCVcoreAg molecules in analyzed solution contained HCVcoreAg, the size of objects on the surface was supposed to grow due to antigen-antibody complex formation. It was demonstrated that the anti-HCVcoreAg height was in the range of (1–1.5) nm (ligand), the HCVcoreAg height was in the range of (1.5–2) nm, height of their complexes was in the range of (3–7) nm, thus the heights of ligand and target proteins were smaller than the height of antibody-antigen complex as was supposed.

AFM image processing and counting of macromolecules and their complexes on the chip surface are carried out using software which allows to recognize the images of objects on the AFM chip, distinguish them by geometrical dimensions and obtain their height distribution.

Biospecific AFM-based fishing can implement reversible or irreversible procedures. In reversible fishing, number of complexes formed on the surface is determined by dissociation constant *Kd*. In irreversible fishing, complexes formed on the surface are additionally covalently cross-linked so that it converts reversible complexing reaction to irreversible one and increase concentration sensitivity of detection of protein complexes due to lifting *Kd* limit.

Using the same ligand-target pair of anti-HCVcore/HCVcoreAg it was shown that irreversible biospecific fishing combined with AFM-based detection of molecules enabled to register HCVcoreAg having four orders less concentration of up to 10^−16^ М in solution compared to the reversible fishing approach [6]. If this registration scheme will be optimized, estimation predicted a capability of further increasing sensitivity by two orders of magnitude. Conversion of reversible bonding into irreversible one increases sensitivity of AFM-based fishing as well as other analytical systems. Detection method of low- and ultralow-abundance proteins in biomaterial can combine concentrating the protein from the sample onto cyanogen bromide-activated Sepharose 4B (via nonspecific binding of free amino groups) and multiple reaction monitoring MS (MRM-MS) [131]. The detection limit and the dependence of the MRM peak areas on the concentration of protein in the sample were determined using the proteins CYP102 and BSA as model system: both proteins in solution and after their addition to human plasma. Nonspecific protein enrichment of proteins from diluted sample volumes of 10–50 mL was found to increase the range of linear dependence of the chromatographic peak area on concentration by more than three orders of magnitude allowing to reach LOD limit (LLOD) as low as 10^−18^ M.

Another challenge for AFM-based molecular detection technique is fishing and registration of target proteins from not only model buffer solution but from biological fluid such as blood serum. This technique was applied for detection of hepatitis C and hepatitis B virus particles in serum [52,130]. After incubation of chip functionalized by anti-HCVcoreAg in the blood serum contained hepatitis C virus, new objects with height in the range of 10–35 nm were imaged using AFM while there were no such objects in the control negative serum. Typical objects in the control experiment had height less than 5 nm. A similar registration scheme was applied for detection of hepatitis B viruses. In this case, objects with height in the range of 10–40 nm were observed after incubation of AFM chip with immobilized antibodies against the hepatitis B virus antigen (HBsAg) in serum. MS confirmed selectivity of proposed analytical system for detection of marker proteins of hepatitis C virus using AFM chips functionalized by antibodies [132,133].

AFM-based fishing can be applied to identification of disease markers of different categories such as infectious and somatic diseases including cancer. Specificity of analysis will be determined by specificity of the ligands (antibodies or aptamers) immobilized on the chip surface. AFM chips functionalized by aptamers (aptamer-based approach) have been applied to carry out AFM-based fishing of envelope glycoprotein gp120 of human immunodeficiency virus (HIV-1) used as biomarker in diagnosis [118]. In aptamer-based approach, the increase of AFM image contrast was observed compared to antibody-based approach when antibodies have been used as bait molecules [118,129,134]. MS analysis allowed to identify the target protein from the chip surface after biospecific fishing that proved aptamer-protein complex formation.

AFM chips having not only one bait spot but a two-dimensional matrix of them are the most promising for diagnostic purposes. Such a matrix of bait spots is a microarray of macromolecules used as baits of proteins associated with different diseases. AFM-based detector combined with the chip assay was implemented to AFM-immunosensor (AFMIS) technology by BioForce (USA) for fast, sensitive and noninvasive detection of viruses [135,136]. The developed platform couples AFM registration with a silicon chip with ultramicrospots of antibodies (20 spots per chip). For multiplex analysis, AFM chip can be also made of mica [137].

To provide a visual orientation and precise cantilever positioning above each spot for further AFM imaging, a method of surface marking was developed on the base of formation the optically visible metallic marks on the AFM chip using magnetron sputtering device [137].

## 5. Conclusions

AFM provides the unique opportunity to study structural and physical characteristics of protein molecules. A wide range of measuring techniques can be combined or used separately. The advantages of AFM include the following: it is non-destructive and high-resolution imaging tool; it does not require complex sample preparation; the ability to operate in air or liquid while obtaining information on a wide range of physical properties of the sample.

Development of the instruments of the latest generation—for instance, BioScope Resolve™ BioAFM [138], which allow simultaneous registration of several signals with independent channels determines the application of AFM as the basis for a novel nanotechnology platform. The use of this platform for simultaneous visualization, determination of stoichiometric and physicochemical properties of biomacromolecules, including the activity of single enzyme molecules, is promising. Introduction of serial high-speed instruments will allow one to employ AFM for solving the tasks of medical diagnostics, and to introduce the method of AFM-based fishing into the wide practice for the detection of medically relevant proteins. The combination of a high-speed scanning and a multiprobe instrument [139] will be promising for the use in proteomic screening.

AFM has great potential when combined with other study approaches. Analysis methods of AFM and mass spectrometry have been combined for visualization and further identification of the protein and protein-protein complexes. AFM-based fishing allows the detection of the low-abundance proteins and characterization of their properties. Such alliances are especially useful to solve problems of proteomics and medical diagnostics.

One of the major disadvantages of AFM have been an impossibility of performing the chemical analysis of a sample. Combining AFM with infrared and other near-field spectroscopy methods can effectively become a technique to overcome that and to obtain data of mechanical properties in relation to chemical analysis at the nanolevel [140,141]. Novel methods are able to retain the high-resolution AFM imaging as well as to analyze a variety of physical, morphological and chemical properties of materials from living tissues to nanotechnology products.
